# Essential Oil from *Pinus Koraiensis* Pinecones Inhibits Gastric Cancer Cells via the HIPPO/YAP Signaling Pathway

**DOI:** 10.3390/molecules24213851

**Published:** 2019-10-25

**Authors:** Yandong Zhang, Chao Xin, Junqiang Qiu, Zhenyu Wang

**Affiliations:** 1 Department of Food Science and Engineering, School of Chemistry and Chemical Engineering, Harbin Institute of Technology, Harbin 150090, China; 15604805165@163.com (Y.Z.); xinchao198811@126.com (C.X.); 2 Department of Inorganic Chemistry and Analytical Chemistry, School of Pharmacy, Hainan Medical University, Haikou 570100, China; qjq000000@163.com

**Keywords:** pinecone, essential oil, gastric cancer, RNA sequencing, HIPPO/YAP signaling pathway

## Abstract

Pinecone is a traditional folk herb, which has been used in China for many years. In this paper, the essential oil from *Pinus koraiensis* pinecones (PEO) was obtained by hydrodistillation and 41 compounds were identified by gas chromatography–mass spectrometry (GC-MS), mainly including α-Pinene (40.91%), Limonene (24.82%), and β-Pinene (7.04%). The purpose of this study was to investigate the anti-tumor activity of PEO on MGC-803 cells and its mechanism. Anti-tumor experiments in vitro showed PEO could significantly inhibit the proliferation and migration of MGC-803 cells, and it also could arrest the cell cycle in the G2/M phase, decrease the mitochondrial membrane potential, and induce apoptosis. Finally, the effects of PEO on genes expression on MGC-803 cells were analyzed by RNA sequencing, and results showed that after treatment with PEO, 100 genes were up-regulated, and 57 genes were down-regulated. According to the KEGG pathway and GSEA, FAT4, STK3, LATS2, YAP1, and AJUBA were down-regulated, which were related to HIPPO signaling pathway. Real-time PCR and western blot further confirmed the results of RNA sequencing. These results indicated that PEO may exert anti-tumor activity via the HIPPO/YAP signaling pathway. The anti-tumor mechanism of this oil can be further studied, which is important for the development of anti-tumor drugs.

## 1. Introduction

Gastric cancer is one of the most common malignancies, with about one million new cases diagnosed each year, ranking third in cancer-related deaths worldwide [1]. Gastric cancer is often diagnosed at a later stage because of the lack of early symptoms, and the frightening is the poor prognosis and limited treatment options [2]. At present, surgical resection, radiotherapy, and chemotherapy are the main methods for the treatment of gastric cancer [3]. Although chemotherapy is a more effective treatment for potential metastatic and metastatic gastric cancer, the currently applied chemotherapy drugs for gastric cancer are difficult to satisfy [4]. In order to improve the side effects and drug resistance of traditional chemotherapy drugs, more and more researches have focused on the development of natural anti-tumor drugs, including natural polyphenols [5], polysaccharides [6], and terpenoids [7].

Essential oils (EOs) have been paid more and more attention due to their unique properties, which play an important role in natural products [8,9]. It has been reported that EOs have many physiological activities, such as antioxidant [10], antibacterial [11], anti-inflammatory [12], anti-tumor [13] and anti-insect [14]. *Pinus koraiensis* is a traditional plant belonging to genus of *Pinus* in the family of *Pinaceae* [15]. Pinecone is an important organ of *Pinus koraiensis*, with the effects of antitussive, expectorant and anti-inflammatory, and it is rich in essential oil. In addition, *Pinus koraiensis* pinecones extracts had a variety of physiological activities such as anti-tumor, anti-angiogenesis, and anti-radiation [16,17,18]. According to reports, pine needle essential oils (from four *Pinus* genera: *p. mugo* subsp. *Mugo*, *P. nigra* subsp. *Nigra*, *P. sylvestris,* and *P. peuce*) had good antimicrobial and insect larvicidal activities [19]. Earlier, the pine needle extract of *Cedrus deodara* was also proven to be a good natural antioxidant [20]. However, there are few reports about *Pinus koraiensis* pinecones essential oil (PEO). Therefore, it was useful for the development of natural anti-tumor drugs to explore the anti-tumor activity of PEO.

HIPPO signaling pathway is a highly conserved cellular signal transduction pathway, which was first found in *Drosophila* [21]. HIPPO pathway controls organ size and cell differentiation by regulating cell proliferation and apoptosis, which is of great significance for the development of tumors [22]. The core of this pathway is a kinase cascade composed of STK3/4-LATS1/2-YAP/TAZ [23]. YAP/TAZ is the main effector of HIPPO pathway, and dephosphorylated YAP/TAZ promotes the transcription of genes regulating proliferation and migration after entering the nucleus [21]. It has been reported that YAP is highly expressed in various tumors, including gastric cancer [24]. More and more studies have proved that YAP can promote the proliferation and metastasis of tumors, which is extremely unfavorable to the prognosis of cancer patients [25,26]. Therefore, it is meaningful to study the potential mechanism of HIPPO/YAP signaling pathway in the proliferation and apoptosis of cancer cells.

In this study, we identified the components of PEO and analyzed its therapeutic potential and mechanism on MGC-803 cells based on RNA sequencing and bioinformatics analysis, which was necessary for the development of anti-tumor drugs.

## 2. Results

### 2.1. Chemical Composition of Essential Oil

**Figure 1:**
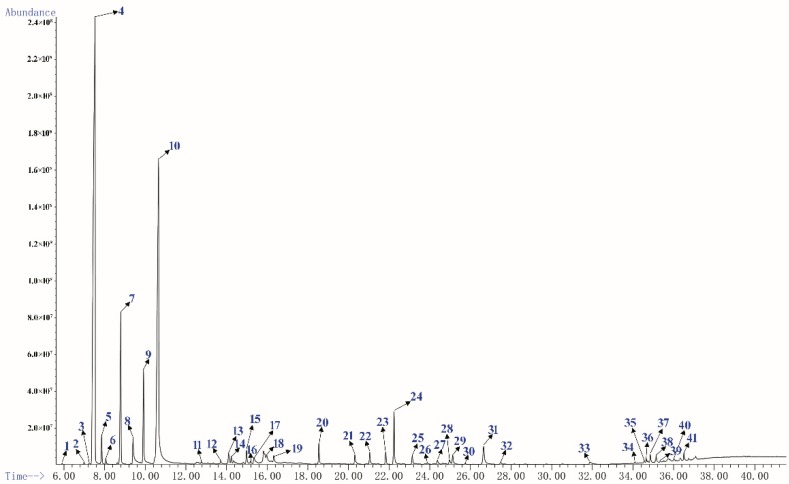
The total ion currency (TIC) profile of PEO.

GC-MS is the most commonly used method for the analysis of essential oils components at present [27,28]. The yellow PEO with characteristic odor was obtained by hydrodistillation and 41 components were identified by GC-MS, accounting 95.73% of the total oil (Table 1), and the main components were respectively α-Pinene (40.91%), Limonene (24.82%), and β-Pinene (7.04%). It was similar to the chemical composition of oils from fresh needles with different pinus species [19,29]. Figure 1 shows sensitive signals in the total ion currency (TIC) profile. The sequence numbers of the peaks in Figure 1 were consistent with those of the compounds in Table 1.

### 2.2. Anti-Tumor Activity of PEO In Vitro

#### 2.2.1. Cell Cytotoxicity Activity In Vitro

**Figure 2:**
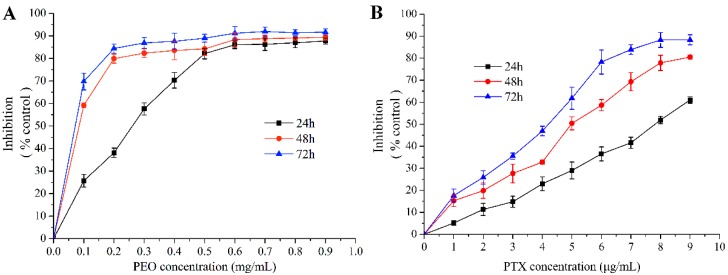
Cytotoxic effects of PEO on MGC-803 cells by MTT assay for 24, 48, and 72 h.

The MTT assay showed that PEO and paclitaxel (PTX) had an obvious cytotoxic activity on MGC-803 cells (Figure 2A,B). As a result, the PEO exhibited a potential growth inhibitory effect on the MGC-803 cells with the EC_50_ were 223.01 ± 5.93 µg/mL at 24 h, 46.17 ± 3.35 µg/mL at 48 h, and 22.36 ± 1.02 µg/mL at 72 h. Under the same conditions, the EC_50_ of PTX were 8.29 ± 0.47 µg/mL at 24 h, 4.44 ± 0.11 µg/mL at 48 h, and 3.22 ± 0.08 µg/mL at 72 h, respectively. These indicated that the PEO had good anti-proliferative activity on MGC-803 cells, and the anti-proliferative activity was gradually enhanced with the increase of concentration and treatment time of essential oil. This was a dose- and time-dependent manner, which was reflected in many medicines.

#### 2.2.2. Cell Migration Capacity Analysis

**Figure 3:**
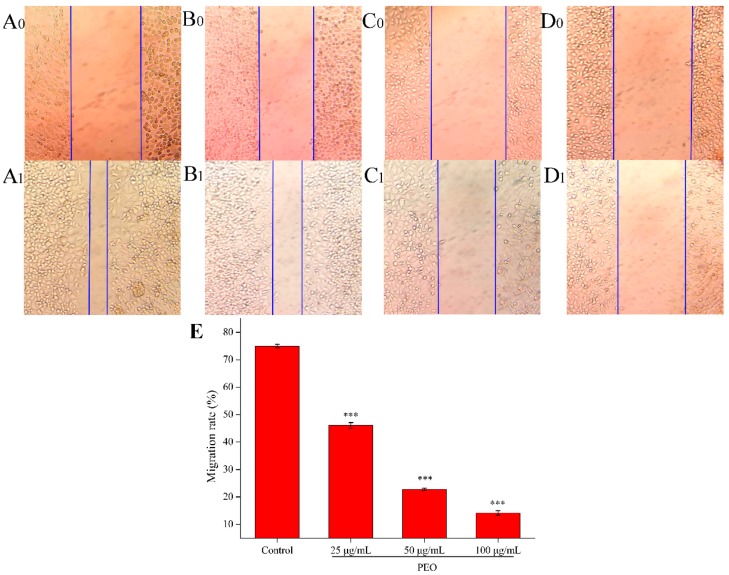
Effects on MGC-803 cell migration capacity. The A–D represented PEO concentrations were 0, 25, 50, and 100 µg/mL, the subscript 0 and 1 that represented the photos (100 ×) were taken after MGC 803 cells exposing to the PEO 0 and 24 h, respectively; E was the migration rates of experimental groups that treated 24 h with PEO compared with 0 h (*** *p* < 0.001).

Cell scratch assay is a simple and rapid method to measure cell migration and repair ability [31]. As shown in Figure 3, compared with the control group, the cell migration ability of the low, middle, and high dose groups decreased significantly (*** *p* < 0.001). The results showed that the PEO could inhibit the migration of MGC-803 cells, and the migration rates were decreased with the increase of the concentration of PEO.

#### 2.2.3. Cell Cycle Analysis

**Figure 4:**
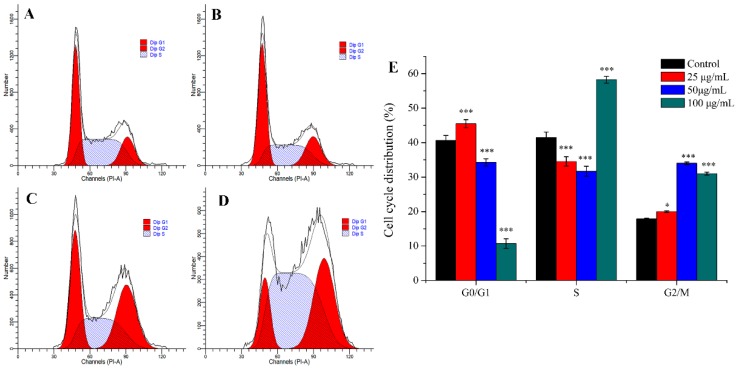
Cell cycle analysis of MGC-803 cells treated by PEO for 24 h. The A–D represented PEO concentrations were 0, 25, 50, and 100 µg/mL, respectively; The E was the numerical histogram of cell cycle distribution (* *p* < 0.05, *** *p* < 0.001).

Many anti-tumor drugs may inhibit the proliferation of tumor cells through cell cycle arrest [32,33]. As shown in Figure 4, PEO led to significant changes of cell cycle progression. Compared with control, when MGC-803 cells were treated with different concentrations of PEO (25, 50, or 100 μg/mL) for 24 h, the percentage of cells in the G2/M phase fraction increased from 17.87% to 19.99%, 34.03%, or 31.01%, respectively. This indicated that PEO can significantly arrest the MGC-803 cells in the G2/M phase (* *p* < 0.05, *** *p* < 0.001).

#### 2.2.4. Cell Apoptosis Analysis

**Figure 5:**
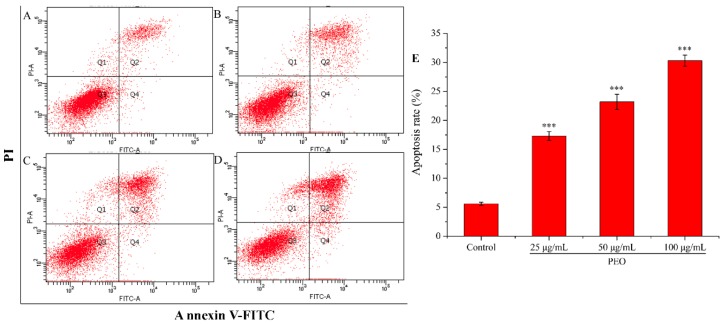
Cell apoptosis analysis of MGC-803 cells treated by PEO for 24 h. The A–D represented PEO concentrations were 0, 25, 50, and 100 µg/mL, respectively; The E was the numerical histogram of apoptosis rate (*** *p* < 0.001).

Apoptosis is the most common way of tumor cells death induced by chemotherapeutic drugs, and it is the main way in which most natural anti-tumor drugs play their roles [34,35]. As shown in Figure 5, the apoptotic rate of the control group was 5.60%, while the three experimental groups were 17.31% (25 μg/mL), 23.22% (50 μg/mL), and 30.30% (100 μg/mL), respectively. Compared with the control group, the apoptotic rates in the three dose groups were significantly different (*** *p* < 0.001), and the apoptotic rates were increased with the increase of PEO concentration. These results demonstrate that PEO can induce apoptosis of MGC-803 cells in a dose-dependent manner.

#### 2.2.5. Analysis of Mitochondrial Membrane Potential

**Figure 6:**
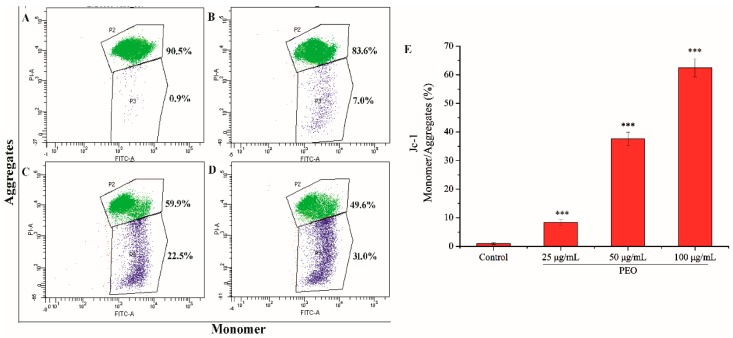
Cell apoptosis analysis of MGC-803 cells treated by PEO for 24 h. The A–D represented PEO concentrations were 0, 25, 50, and 100 µg/mL, respectively; The E was the numerical histogram of the ratio of JC-1 monomer to aggregates (*** *p* < 0.001).

The mitochondrial membrane potential of MGC-803 cells was decreased significantly after being treated by PEO (Figure 6). As shown in Figure 6A–D, with the increase of PEO concentration, the number of JC-I monomer was increased from 0.9% to 31.0%. Compared with the control group, the rates of JC-I monomer/aggregate in the three dose groups were significantly different (Figure 6E, *** *p* < 0.001). These further indicated that PEO can induce apoptosis of MGC-803 cells.

### 2.3. RNA Sequencing Analysis

#### 2.3.1. Screening for Differentially Expressed Genes (DEGs)

**Figure 7:**
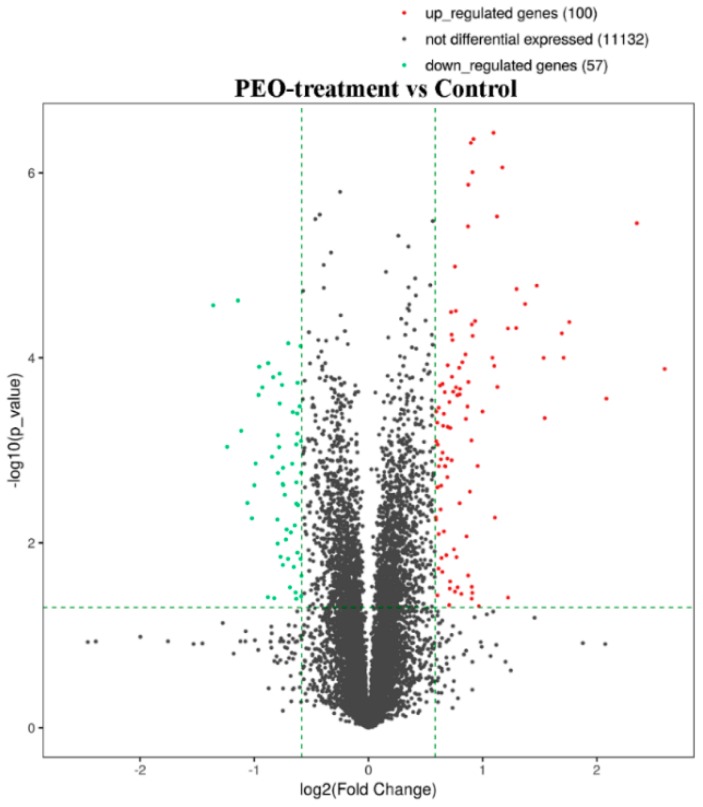
The volcano plot of mRNA. The vertical two green lines were up-regulated (right side) and down-regulated (left side), respectively. The green parallel lines corresponded to the p-value threshold. Green dots represent down-regulated genes with significant differences, red dots represent up-regulated genes with significant differences, and gray dots represent non-significant different genes.

**Figure 8:**
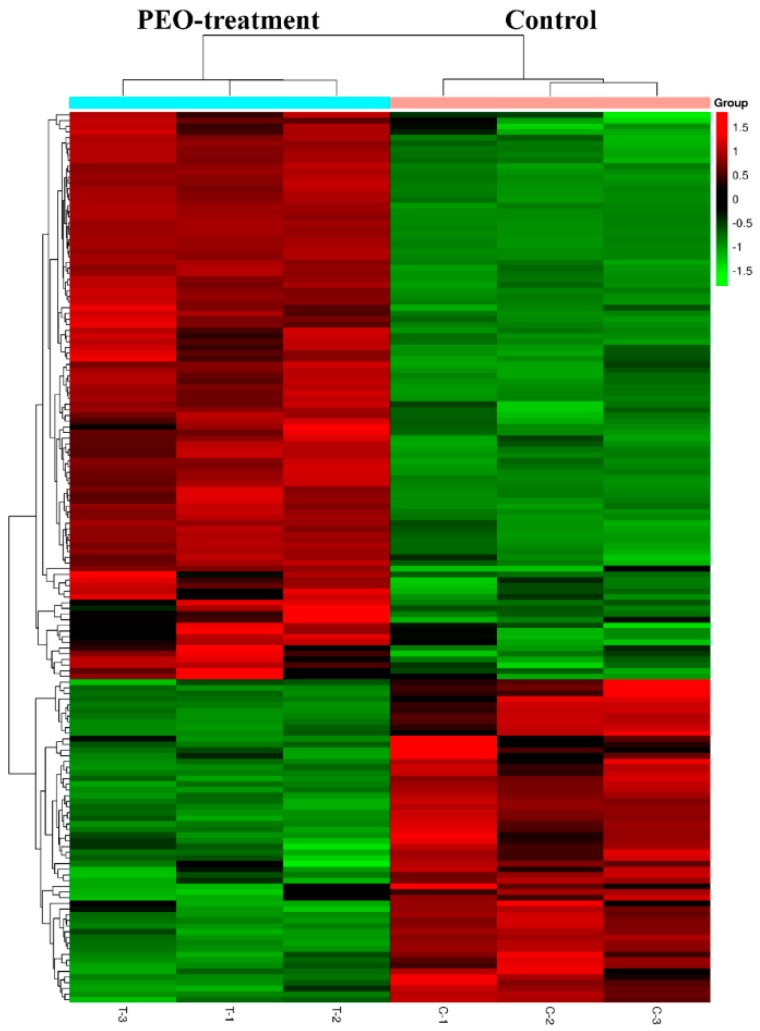
Heat map of DEGs with fold change expression >1.5. MGC-803 cells were treated with 50 µg/mL PEO or control for 24 h (*n* = 3). Rows represent genes and columns represent samples. Red blocks represent high and green blocks low expression relative to comparison cells.

After treatment of MGC-803 cells with 50 µg/mL PEO for 24 h, RNA sequencing analysis indicated that there were 157 differentially expressed genes (100 up-regulated and 57 down-regulated) in the experimental groups compared with the control (Figure 7 and Figure 8). The top 10 differentially expressed genes were presented in Table 2.

#### 2.3.2. Functional and Pathway Enrichment Analyses

**Figure 9:**
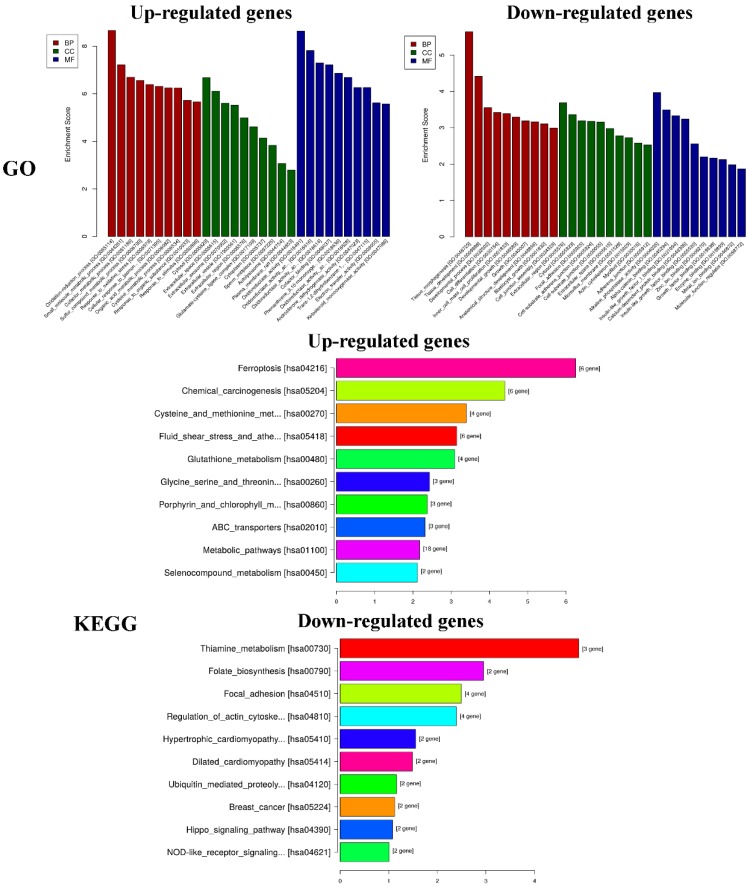
Results of functional and pathway enrichment analyses. GO: Gene ontology, KEGG: Kyoto encyclopedia of genes and genomes.

GO analysis of DEGs was performed to determine the main functions of PEO on MGC-803 cells inhibition. The top 10 GO items (BP, CC, MF) and KEGG pathways with the most significant difference in the results was shown in Figure 9. According to the results of KEGG pathway and GSEA, we found that the changes of HIPPO signaling pathway were the most significant in cancer-related signaling pathways (experimental groups versus control groups). The genes of FAT4, STK3, LATS2, YAP1, and AJUBA were all down-regulated (* *p* < 0.05). Among them, YAP1 is the crucial effector of HIPPO signaling pathway, and plays a key role in HIPPO signaling pathway [36]. We speculated that the PEO inhibited the proliferation of MGC-803 cells maybe by regulating the HIPPO signaling pathway. Next, we verified the difference of FAT4, STK3, LATS2, YAP1, and AJUBA genes expression on MGC-803 cells treated with PEO or control by real-time PCR.

#### 2.3.3. Real-Time PCR Analysis

**Figure 10:**
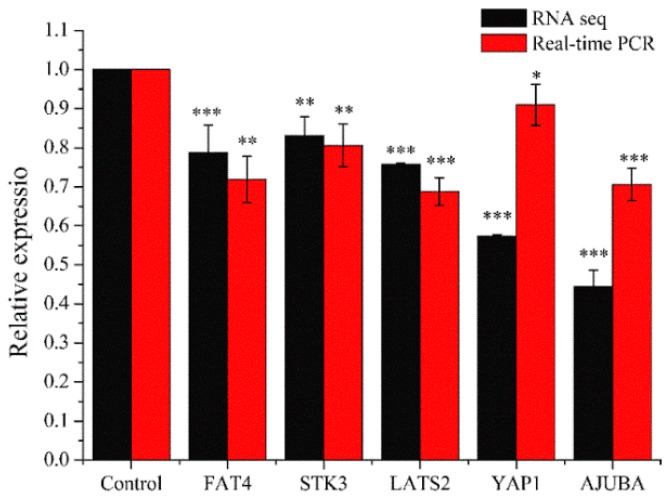
Results of real-time PCR. MGC-803 cells were treated with 50 µg/mL PEO or control for 24 h (* *p* < 0.05, ** *p* < 0.01, *** *p* < 0.001, *n* = 3).

Real-time PCR was performed for genes quantitative analysis to confirm the changes of genes in RNA sequencing results. We selected five genes (FAT4, STK3, LATS2, YAP1, and AJUBA) which were related to HIPPO signaling pathway. As shown in Figure 10, the relative expression in each gene (FAT4, STK3, LATS2, YAP1, or AJUBA) were similar between real-time PCR and RNA Sequencing. Compared with the control group, the expression levels in the experimental groups were significantly down-regulated (* *p* < 0.05, ** *p* < 0.01, *** *p* < 0.001). This showed that RNA Sequencing was properly performed and the PEO could regulate HIPPO signaling pathway.

#### 2.3.4. Western Blot Analysis

**Figure 11:**
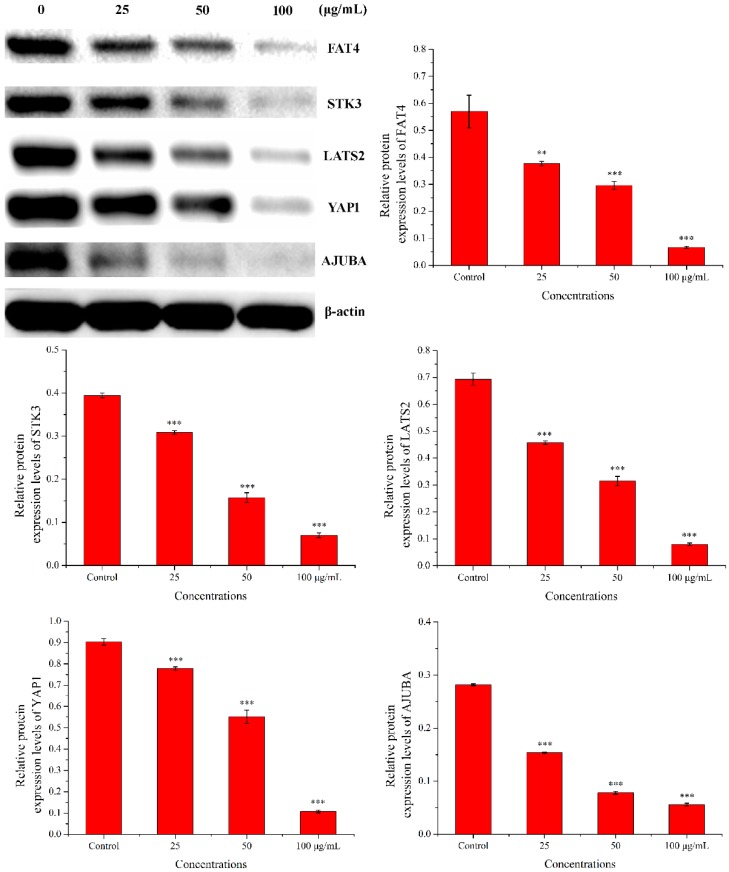
Results of western blot. MGC-803 cells were treated with 0, 25, 50, and 100 µg/mL PEO for 24 h (*n* = 3, ** *p* < 0.01; *** *p* < 0.001).

Western blot was used to check the expression of five proteins (FAT4, STK3, LATS2, YAP1, and AJUBA). As shown in Figure 11, compared with the control group, the expression levels of FAT4, STK3, LATS2, YAP1, and AJUBA proteins were significantly down-regulated in a dose-dependent manner when treated with PEO for 24 h (* *p* < 0.05, ** *p* < 0.01, *** *p* < 0.001). The results were consistent with the RNA sequencing and real-time PCR.

## 3. Discussion

EOs are an important source of natural antioxidant, antibacterial, anti-inflammatory, and anti-tumor agents, and have received more and more attention [37,38,39]. The chemical constituents and relative contents of EOs are not only related to raw materials, but also to extraction methods [40]. EO is a mixture of various small molecule compounds that exerts its physiological activities as a combination drug. For example, Galdino et al. [41] reported that the essential oil derived from *Spiranthera odoratissima* leaves and its main component (β-caryophyllene) had anxiolytic-like effects, but their effects and mechanisms were completely different. Therefore, it is difficult to determine whether a component plays a leading role, whether it is the most abundant or not. Nevertheless, the difference in composition and content is still an important factor affecting the quality of EOs [42].

As a result, terpenes were the main components of PEO extracted by hydrodistillation, mainly including α-Pinene (40.91%) and Limonene (24.82%). It is reported that both α-Pinene and Limonene had anti-tumor activity, which can inhibit the proliferation of a variety of human tumor cells, including prostate cancer, breast cancer, liver cancer, and gastric cancer cells [43,44]. Chen et al. [45] found that α-Pinene had good anti-tumor activity in vitro and in vivo, and it can inhibit the proliferation of BEL-7402 cells by arresting the cell cycle in the G2/M phase. However, the anti-tumor activity of Limonene is weak, and it is usually necessary to synthesize Limonene derivatives to enhance its anti-tumor activity [46]. In this study, the anti-tumor activity experiments showed that PEO could significantly inhibit the proliferation and migration of MGC-803 cells, arrest cell cycle in G2/M phase, and finally induce apoptosis (*p* < 0.05). These results proved that PEO had good anti-tumor activity, similar to many EOs that had been reported [47,48].

Most of the reports were based on the above experiments to prove the anti-tumor activity of EOs, but the anti-tumor mechanism was not very clear. This was closely related to the complex pathogenesis of tumors. However, the wide application of RNA sequencing technology facilitated the study of the mechanism of anti-tumor drugs [49]. RNA sequencing is currently the preferred technology for gene expression analysis, and it could quickly and accurately detect the differences of gene expression among different groups [50]. In this study, RNA sequencing revealed 157 differentially expressed genes in the PEO-treated groups compared with the control groups (*p* < 0.05). According to the KEGG pathway and GSEA analysis, HIPPO signaling pathway is the key to the anti-tumor effect of PEO on MGC-803 cells. As a result, genes associated with HIPPO signaling pathways were significantly down-regulated, including FAT4, STK3, LATS2, YAP1, and AJUBA (*p* < 0.05).

HIPPO signaling pathway is an acknowledged anti-tumor pathway, which has been widely studied [22]. FAT4 is a multiple upstream signal input factor of HIPPO signaling pathway, and STK3 and LATS2 are crucial in the core kinase cascade. YAP1 is the effector of Hippo signaling pathway with important functions in cell proliferation, apoptosis, invasion, and migration [51]. AJUBA function as negative regulators of the Hippo pathway, affecting cell proliferation and controlling tissue size [52]. The central axis of the pathway comprises a phosphorylation cascade of STK3/4-LATS1/2-YAP/TAZ in humans, and it plays an important role in the development of tumors [53].

YAP is identified as a well-characterized human oncogene and it is highly expressed in several types of cancers including gastric [54]. Our results demonstrated that PEO can down-regulate the expression of YAP1, which may be the reason why PEO inhibits the proliferation and migration of MGC803 cells and promoting apoptosis. In addition, AJUBA LIM proteins interact with LATS/Wts and WW45/Sav to inhibit phosphorylation of YAP1 and up-regulates Cyclin E and DIAPI, thereby promoting cell proliferation [55]. In this study, the down-regulation of LATS2 and AJUBA may lead to the enhancement of YAP1 phosphorylation, decrease its activity in the nucleus and inhibit the proliferation of MGC-803 cells. As the study of Li et al. [56], AJUBA overexpression inhibited HIPPO signaling by upregulating YAP protein expression and promotes proliferation in gastric cancer cells. This is consistent with the results of this experiment.

## 4. Materials and Methods

### 4.1. Plant Materials and Oil Extraction

The fresh pinecones of *Pinus koraiensis* were obtained from Yichun Hongxing District Forestry Bureau (Yichun, China), in October 2018. The air-dried pinecones were grinded into powder (particle size < 840 µm) and treated with hydrodistillation in a Clevenger-type apparatus for 4 h. The PEO was stored at −20 °C until experimental studies.

### 4.2. GC/MS Analysis

GC/MS analysis was performed by a gas chromatography (6890N, Agilent Technologies, Santa Clara, CA, USA) coupled with a mass spectrometer (5975N, Agilent Technologies, Santa Clara, CA, USA) operating at 70 eV ionization energy equipped with a DB-5-MS capillary column (30 m × 0.25 mm × 0.25 µm). The injector and detector temperatures were maintained at 280 °C. The temperature timeline of the oven was programmed as follows: The initial temperature was set to be 40 °C and was then allowed to increase to 150 °C. Finally, the temperature increased to 310 °C kept for 5 min. 1 mL of PEO was dissolved in n-hexane and injected; and the split mode was 1:50. Helium was used as the carrier gas with a flow rate of 1.0 mL/min. Then, the MSD Chem Station Software (AMDIS-32, Agilent Technologies, Santa Clara, CA, USA) was used to handle mass spectra and chromatograms. Most constituents were tentatively identified by comparison of their retention indices (RIs), established in accordance with reference to a homologous series of C8−C40 n-alkanes that had been injected following the injection of the PEO under the same chromatographic conditions. The component identification of PEO was performed by comparing retention indices and matching the recorded mass spectra of each compound with NIST 17 Mass Spectral libraries.

### 4.3. In Vitro Anti-Tumors Assay

#### 4.3.1. Cytotoxicity Assay

The MGC-803 cell line was obtained from Cancer Hospital Affiliated to Harbin Medical University (Harbin, China). Cytotoxicity assay was determined by MTT assay, the experimental steps referred to the previous reports [6,57]. The concentration of DMSO in the medium was kept in 0.1% (*w*/*v*). The MGC-803 cells (1~5 × 10^4^) were cultured in 96-well plates and treated with a various of PEO concentrations (0.1 ~ 0.9 mg/mL) for 24, 48, and 72 h. 10 µL MTT solution (5 mg/mL) was added to cells and incubated for 4 h at 37 °C. Then, the medium was removed and 150 µL DMSO was added to cells. The absorbance was determined at 490 nm. Paclitaxel (Aladdin, Shanghai, China) was used as a positive control drug. All experiments were undertaken in sextuplicate. The cell proliferation activity was calculated as follows: Inhibition rate (%) = (A_0_ − A_1_)/A_0_ × 100, A_1_ represented the absorbance of the experimental groups, while A_0_ represented the absorbance of the control group.

#### 4.3.2. Scratch Wound-Healing Assay

Cell scratch assay was performed as previously reported [58]. The logarithmic phase MGC-803 cells were grown in a 6-well plate with appropriate density until cells covering the whole dish and starved overnight in DMEM media with 1% fetal bovine serum (Sigma-Aldrich, St. Louis, MO, USA). After making the linear scratch, the medium was removed and plates were washed three times with PBS. The cells were treated with various concentrations of PEO (0, 25, 50, and 100 µg/mL) for 24 h. The medium was discarded and photos were taken immediately under an inverted phase contrast microscope selecting 10 fields. The width of the scratched area was measured by Image J software.

#### 4.3.3. Cell Cycle Analysis

The MGC 803 cells (1~5 × 10^5^) in the logarithmic phase were cultured in a 6-well plate at 37 °C, 5% CO_2_ (*v*/*v*) for 10 ~12 h. After treating MGC-803 cells with PEO at different concentrations (0, 25, 50, and 100 µg/mL) for 24 h, cells were centrifuged and stained with 50 µg/mL PI and 100 µg/mL RNase A for 30 min in the dark at room temperature. All operations were carried out in accordance with the requirements of the cell cycle detection kit (Beyotime Biotechnology, Shanghai, China). The samples were analyzed by flow cytometer, and the data were analyzed by using the ModFit LT software (Verity Software House, Topsham, ME, USA) [59].

#### 4.3.4. Apoptosis Analysis

All operations were carried out in accordance with the requirements of the Annexin V-FITC/PI Kit (4A Biotech Co., Ltd., Beijing, China). The logarithmic phase MGC-803 cells (1~5 × 10^5^) were grown in a 6-well plate culturing for 12 h, then the cells were treated with various concentrations of PEO (0, 25, 50, and 100 µg/mL) for 24 h. After cells were centrifuged and the medium was discarded, cells were washed with the pre-cooled PBS. A total of 1~5 × 10^6^ cells were resuspended in the Binding Buffer, then added in 5 µL Annexin V/FITC and incubated for 3 min at room temperature in the dark. Finally, 10 µL and 20 µg/mL PI were added and the samples were analyzed by flow cytometer immediately.

#### 4.3.5. Measurement of the Mitochondrial Membrane Potential (ΔΨm) with JC-1

The Mitochondrial membrane potential of MGC-803 cells treated with PEO was measured by JC-1 dye (Solarbio, Beijing, China). The MGC-803 cells in the logarithmic phase were cultured in a 6-well plate at 37 °C, 5% CO_2_ (*v*/*v*) at a density of 3 ~ 5 × 10^5^ cells per well. After 24 h of treatment with different concentrations PEO (0, 25, 50, and 100 µg/mL), cells were centrifuged and collected. 1 mL Jc-1 reagent (the concentration of Jc-1 was 10 µg/mL) was added to each centrifuge tube and incubated for 20 min at 37 °C. Then, the samples were rinsed twice with PBS and analyzed by flow cytometer.

### 4.4. The Mechanism of PEO on MGC-803 Cells

#### 4.4.1. RNA Extraction and Sequencing

After the treatment with PEO for 24 h, total RNA was extracted using TRIzol reagent (Thermo Fisher Scientific, Waltham, MA, USA) and the RNeasy kit (Qiagen, Hilden, Germany) according to the manufacturer’s instructions. The integrity of the total RNA was checked by agarose gel electrophoresis, and NanoDrop ND-1000 was used for quantitative and further quality control. A total of six samples were available, including three samples of test groups (T-1, T-2, and T-3) and three samples of control groups (C-1, C-2, and C-3). 1~2 µg total RNA was selected from each sample for sequencing library construction. The mRNA of total RNA was enriched using the NEB Next^®^ Poly(A) mRNA Magnetic Isolation Module (New England Biolabs, USA) (or the rRNA was removed using RiboZero Magnetic Gold Kit, Epicentre, Charlotte, NC, USA). After treatment, the product RNA was constructed by the Library using KAPA Stranded RNA-Seq Library Prep Kit (Illumina, San Diego, CA, USA). The constructed library was checked by Agilent 2100 Bioanalyzer (library concentration, fragment size 400–600 bp, whether there were junctions, etc.) and the final quantification of the library was carried out by qPCR. The clustering of the index-coded samples was performed on a cBot Cluster Generation System by using TruSeq SR Cluster Kit v3-cBot-HS (#GD-401-3001, Illumina, San Diego, CA, USA) according to the manufacturer’s instructions. After cluster generation, the libraries were sequenced on an Illumina Hiseq 4000 (Illumina, San Diego, CA, USA) and 150 bp paired-end reads were generated. Accession numbers: GSE137480 [NCBI tracking system #20316023].

#### 4.4.2. Differential Gene Expression Analysis

Ballgown was used to quantify the expression level of samples and to analyze the differential expression of genes between the control and the experimental groups. The criteria for this analysis were FPKM (Fragments per kilobase of transcript per million mapped reads) ≥ 0.5 and *p* value ≤ 0.05.

#### 4.4.3. Gene Ontology (GO) and Pathway Analysis

GO is a gene functional classification entry that includes three sub-items of molecular function, cellular composition, and involved biological processes. We used GO analysis to determine which specific functional items are most directly associated with the DEGs. Pathway analysis was used to determine the significant pathways in which DEGs participate.

#### 4.4.4. Real-Time PCR Analysis

DNA amplification and detection were performed with a ViiA 7 Real-time PCR System (Applied Biosystems) to confirm the RNA-seq result. The full primer list is shown in Supplementary and Table 3. The real-time polymerase chain reaction (real-time PCR) amplifications were carried out in a total reaction volume of 10 µL, containing 2 µL of DNA extract. The reaction mixture contained 2.0 µL water (Roche, Mannheim, Germany), 5 μL of master mix 2 × (Arraystar, Rockville, MD, USA), and 0.5 µL of each primer (10 µM). Real-time PCR was performed with the following conditions: 95 °C/10 min followed by 40 cycles of 95 °C/10 s, 60 °C/60 s. Finally, a melting curve was performed by heating 95 °C/10 s, 60 °C/60 s, 95 °C/15 s, and heating again from 60 to 99 °C.

#### 4.4.5. Western Blot Analysis

The experimental steps referred to the previous reports [57]. MGC-803 cells were collected after treatment with PEO (0, 25, 50, and 100 μg/mL) for 24 h and then lysed in an ice-cold RIPA buffer (Aladdin Industrial Corporation, Shanghai, China). After centrifugation at 12,000 rpm for 10 min, the protein in the supernatant was quantified by bicinchoninic acid (BCA) protein assay kit (Beyotime Co, Shanghai, China). The proteins in each sample were separated via SDS polyacrylamide on a 10% gel and transferred to PVDF membrane. Blocked with 5% non-fat milk for 1 h at room temperature, then the membranes were incubated with β-actin, FAT4 (ABGENT, San Diego, CA, USA), STK3, LATS2, YAP1, and AJUBA antibodies (WUHAN SANYING, Wuhan, China) overnight at 4 °C, washing with TBST, and then incubated with HRP-conjugated secondary antibodies for 1 h at room temperature. Proteins were detected by ECL procedures (Thermo Fisher Scientific, Waltham, MA, USA) and analyzed by Image J software. The experiments were repeated three times.

### 4.5. Statistical Analysis

All experiments were repeated three times, and the experimental data were presented in the form of mean ± SD. One-way analysis of variance (ANOVA) were used to analyze significant differences. *p* < 0.05 was considered to indicate a statistically significant result. The criterion for statistically significant results was *p* < 0.05.

## 5. Conclusions

The experimental results show that PEO had good anti-tumor activity on MGC-803 cells. The results of RNA sequencing showed that the related genes FAT4, STK3, LATS2, YAP1, and AJUBA of HIPPO signaling pathway had significant changes, which were verified by real-time PCR and Western blot experiments. These results suggested that HIPPO signaling pathway was indeed involved in the anti-tumor activity of PEO on MGC-803 cells, but these did not prove that HIPPO signaling pathway was the only pathway in the anti-tumor mechanism. This study provided a theoretical basis and research direction for the next study on the anti-tumor mechanism of PEO. It is useful for the development of natural anti-tumor drugs and the comprehensive utilization of *Pinus koraiensis* resources.

## Figures and Tables

**Table 1:** GC-MS analysis results of PEO components.

No.	Compounds	RI ^a^	RI ^b^	%
1	Santene	882	884	0.06
2	Tricyclene	918	919	0.11
3	Bicyclo [3.1.0]hexane, 4-methyl-1-(1-methylethyl)-didehydro deriv.	926	—	0.11
4	α-Pinene	934	934	40.91
5	Camphene	944	945	1.08
6	Cosmene	968	—	0.07
7	β-Pinene	973	972	7.04
8	β-Myrcene	992	992	2.04
9	3-Carene	1008	1007	4.12
10	Limonene	1030	1031	24.82
11	Bicyclo [3.1.0]hexane, 6-isopropylidene-1-methyl-	1088	—	0.24
12	L-trans-Pinocarveol	1138	1140	0.58
13	L-camphor	1142	1142	0.35
14	*cis*-Verbenol	1145	1137	0.24
15	Pinocarvone	1161	1162	0.13
16	Borneol	1166	1160	0.64
17	3-Pinanone	1173	—	0.15
18	4-Terpineol	1178	1177	0.59
19	p-menth-1-en-8-ol	1193	1192	1.41
20	Myrtenol	1197	1195	0.80
21	Verbenone	1209	1205	0.58
22	L-α-bornyl acetate	1285	1287	0.92
23	α-Longipinene	1349	1350	0.55
24	Copaene	1375	1375	0.57
25	D-longifolene	1404	1405	0.59
26	β-Caryophyllen	1419	1420	2.57
27	α-Caryophyllen	1454	1452	0.43
28	γ-Muurolene	1477	1478	0.06
29	Germacrene D	1482	1484	0.11
30	α-Muurolene	1501	1502	0.26
31	α-Himachalene	1510	1500	0.07
32	(+)-δ-Cadinene	1523	1526	0.29
33	Caryophyllene oxide	1583	1585	1.48
34	α-Humulene oxide II	1610	1608	0.12
35	Pentyl cinnamate	1760	—	0.27
36	Cembrene	1941	1944	0.06
37	1-Heptatriacotanol	1956	—	0.02
38	4b,8-Dimethyl-2-isopropylphenanthrene, 4b,5,6,7,8,8a,9,10-octahydro-	1995	—	0.48
39	Manoyl oxide	2009	—	0.17
40	7-Isopropyl-1,1,4a-trimethyl-1,2,3,4,4a,9,10,10a-octahydrophenanthrene	2079	—	0.34
41	1-Phenanthrenecarboxaldehyde, 1,2,3,4,4a,9,10,10a-octahydro-1,4a-dimethyl-7-(1-methylethyl)-, [1R-(1. α, 4a.β,10a.α)]-	2316	—	0.30
	Total			95.73

^a^ Retention index determined on a DB-5-MS capillary column relative to a series of n-alkanes (C8–C40). ^b^ Retention index reported from the literature [19,29,30].

**Table 2:** Top 10 differentially expressed genes in MGC-803 cells (PEO treatment versus control).

Rank	Gene Name	Gene Feature	log2fc	Fold Change	*p*-Value
1	SLC7A11	Up-regulated	2.35	5.10	3.50 × 10^−6^
2	A2M	Down-regulated	−1.14	0.45	2.40 × 10^−5^
3	ALPP	Down-regulated	−1.36	0.39	2.71 × 10^−5^
4	HTRA3	Up-regulated	1.76	3.38	4.11 × 10^−5^
5	AKR1C2	Up-regulated	1.71	3.27	9.99 × 10^−5^
6	AKR1C1	Up-regulated	2.59	6.04	1.32 × 10^−4^
7	HMOX1	Up-regulated	2.08	4.24	2.76 × 10^−4^
8	AC069257.3	Down-regulated	−1.11	0.46	6.14 × 10^−4^
9	ALPI	Down-regulated	−1.24	0.42	9.15 × 10^−4^
10	CA9	Down-regulated	−1.06	0.48	3.71 × 10^−3^

**Table 3:** The primers used for real-time PCR analysis.

Gene	Bidirectional Primer Sequence
GAPDH (HUMAN)	F:5′ GGGAAACTGTGGCGTGAT 3’ R:5′ GAGTGGGTGTCGCTGTTGA 3’
LATS2	F:5’ TGGTGGAGTGTTGGAGTGAT 3’ R:5′ AGCGTGTTCTCCCAGTTGAT 3’
YAP1	F:5’ GCCAGCAGGTTGGGAGAT 3’ R:5′TGTGATTTAAGAAGTATCTCTGACC 3’
AJUBA	F:5’ TGTCACCGACTACCACAAA 3’ R:5′ ATCACCCTCACGATGTCC 3’
FAT4	F:5’ ATTTAGGACCAGAAGCGAGAA 3’ R:5′ CTCTCCACTTTCCCAGCAA 3’
STK3	F:5’ TCAAGAATGCCAAACCTGTA 3’ R:5′ GGATTCCAACATCGTGCTA 3’

## Supplementary Materials

The full primer list is available as online at https://www.mdpi.com/1420-3049/24/21/3851/s1.
