# Supplementary figures and images for: Essential Oil from Pinus Koraiensis Pinecones Inhibits Gastric Cancer Cells via the HIPPO/YAP Signaling Pathway
Source: Molecules. 2019 Oct 25;24(21):3851. doi: 10.3390/molecules24213851 (PMC6864528; doi:10.3390/molecules24213851)

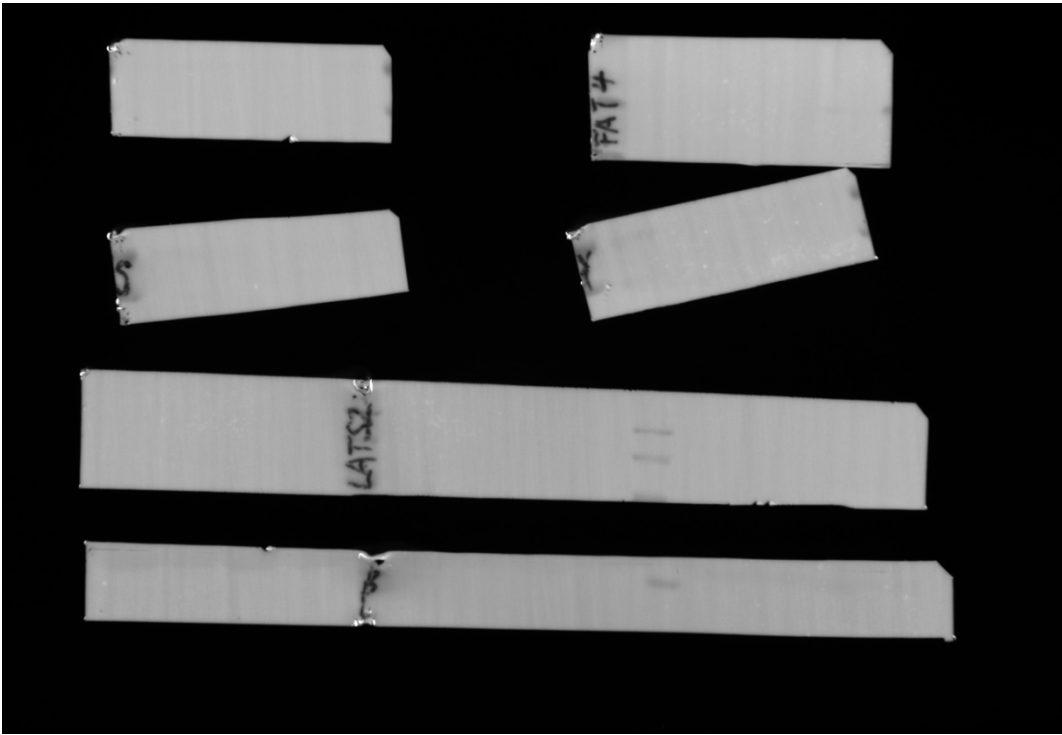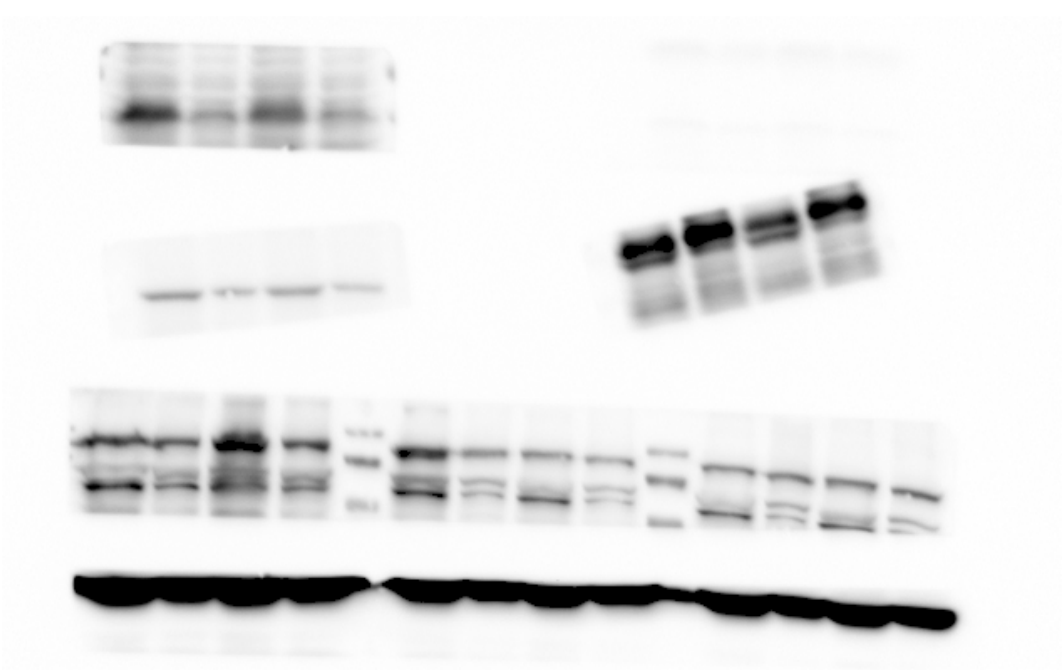

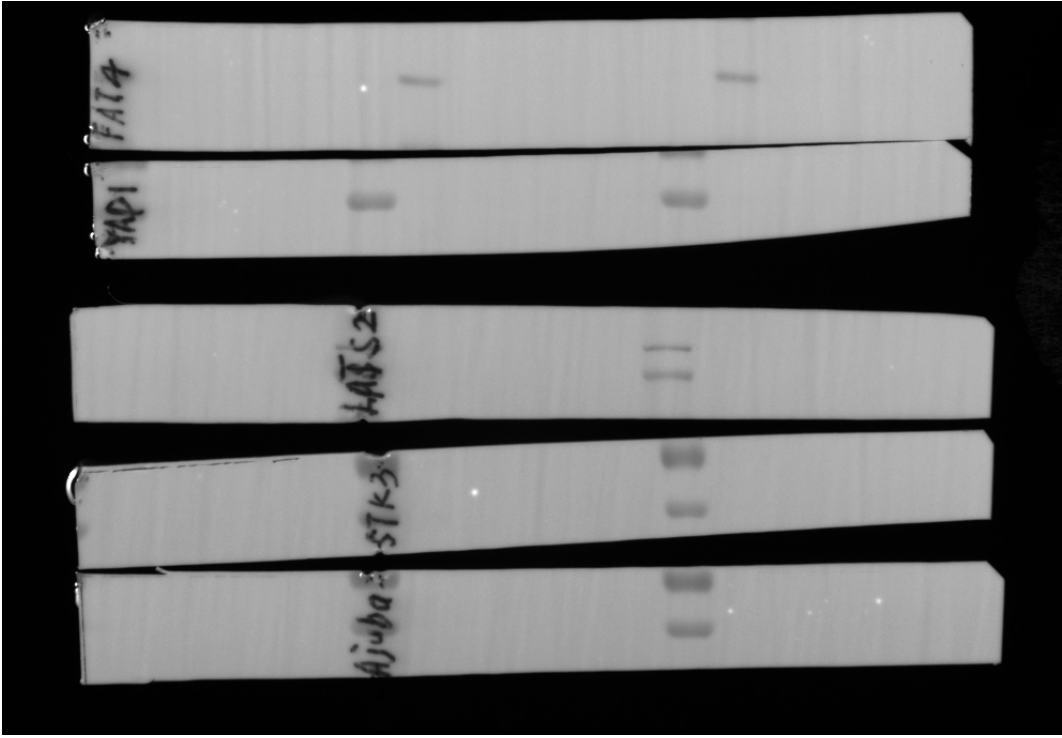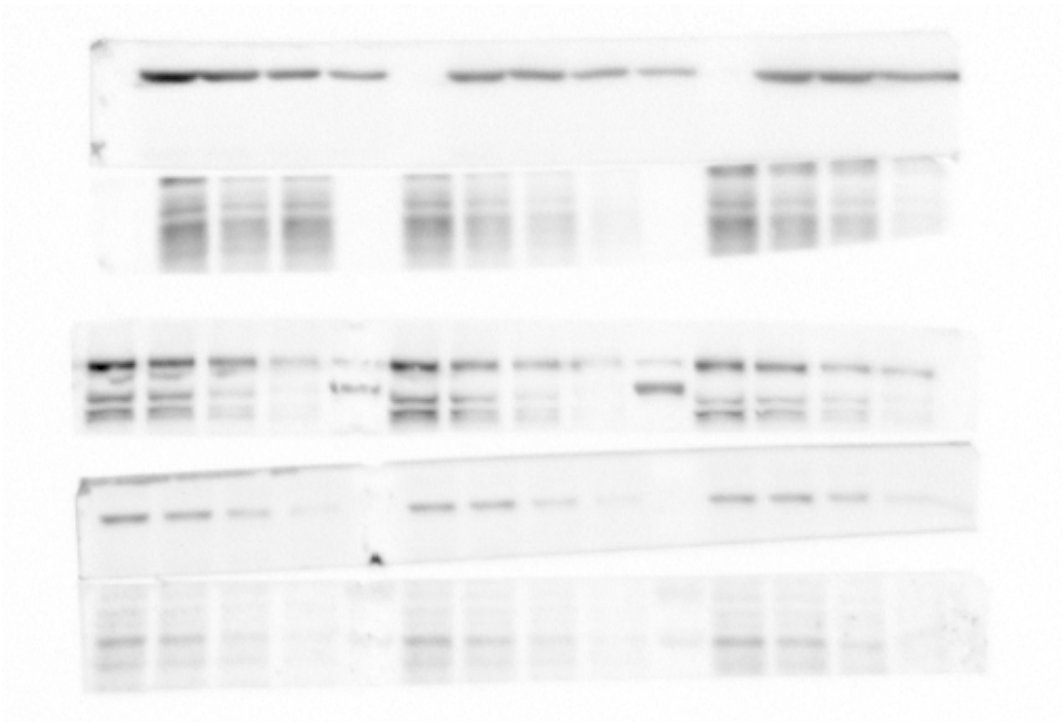

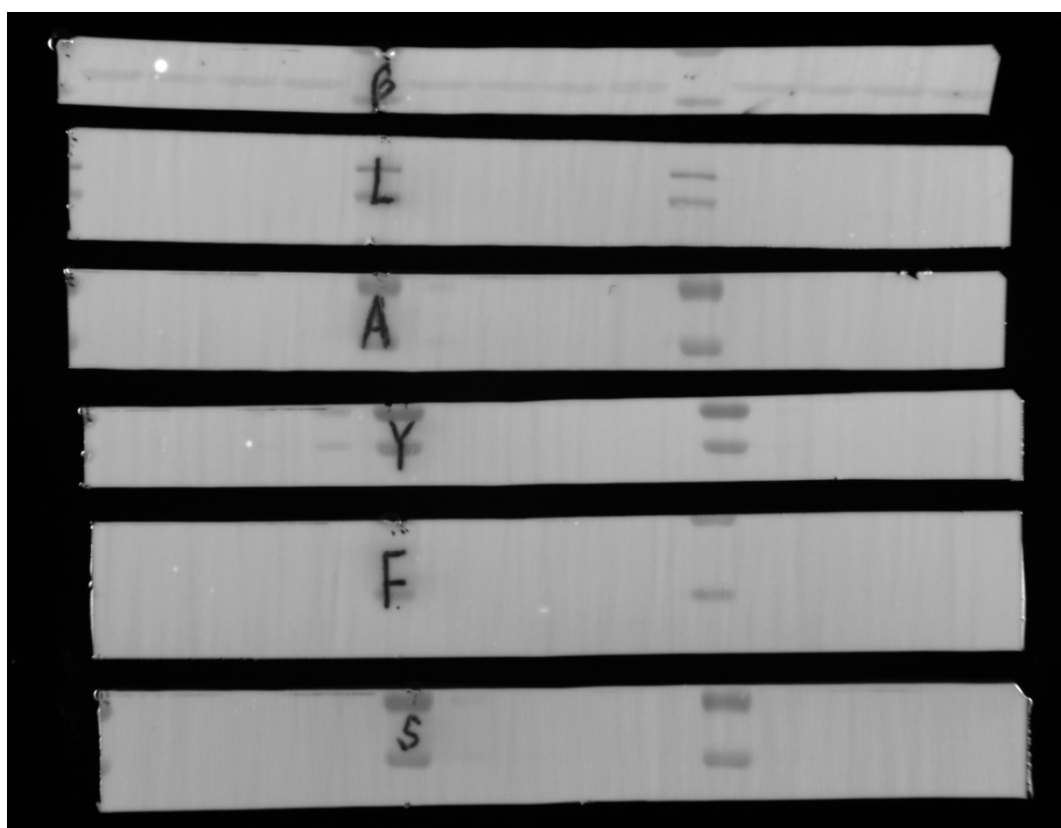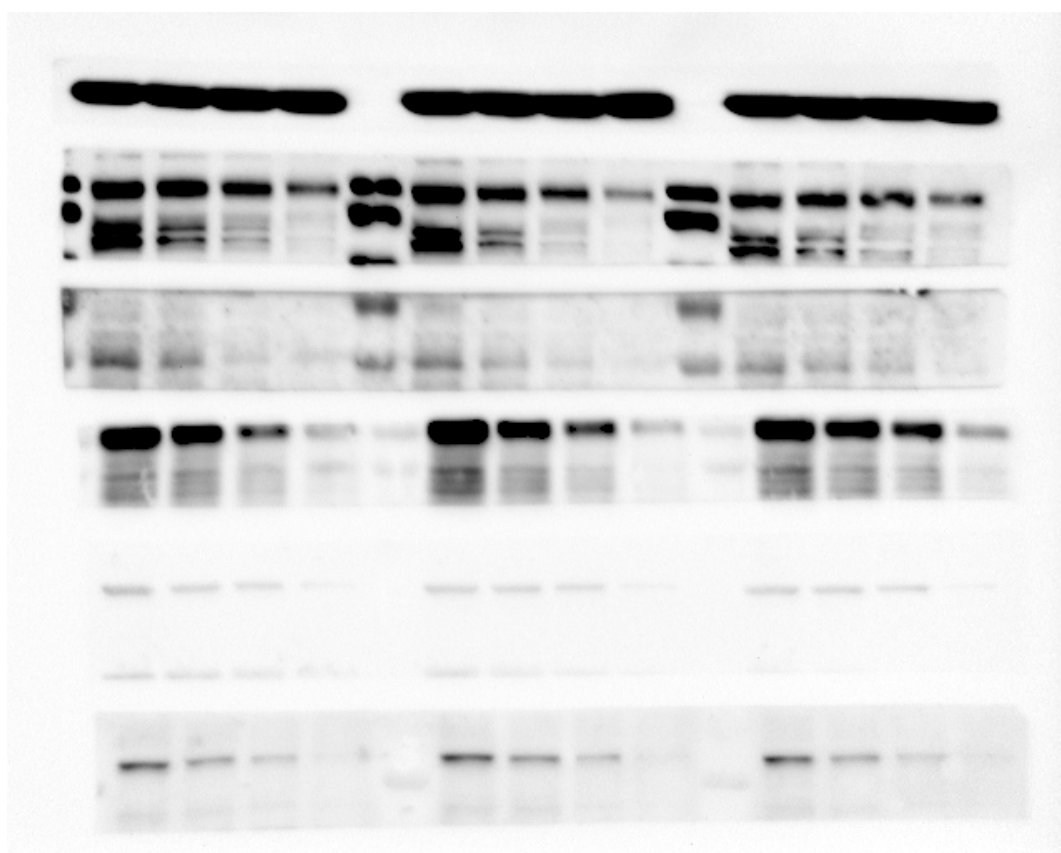

Supplement: Supplementary file 1 [file molecules-24-03851-s001.pdf]
